# Trends of mortality rate in patients with congenital heart defects in Germany—analysis of nationwide data of the Federal Statistical Office of Germany

**DOI:** 10.1007/s00392-023-02370-6

**Published:** 2024-03-04

**Authors:** Hashim Abdul-Khaliq, Delphina Gomes, Sascha Meyer, Rüdiger von Kries, Stefan Wagenpfeil, Jochen Pfeifer, Martin Poryo

**Affiliations:** 1 Department of Pediatric Cardiology, Saarland University Medical Center, Kirrberger Straße, 66421 Homburg/Saar, Germany; 2 Competence Network for Congenital Heart Defects, DZHK (German Centre for Cardiovascular Research), Berlin, Germany; 3 Institute of Social Pediatrics and Adolescent Medicine, Division of Pediatric Epidemiology, Ludwig-Maximilians-University Munich, Munich, Germany; 4 Department of Pediatrics and Neonatology, Section of Intensive Care, Saarland University Medical Center, Homburg/Saar, Germany; 5 Institute for Medical Biometry, Epidemiology and Medical Informatics, Saarland University Medical Center, Homburg/Saar, Germany

**Keywords:** Congenital heart defect, Mortality, Newborn, Infant

## Abstract

**Background:**

Congenital heart defects (CHD) are still associated with an increased morbidity and mortality. The aim of this study was to analyze trends of mortality rates in patients with CHD between 1998 and 2018 in Germany.

**Methods:**

Data of registered deaths with an underlying diagnosis of CHD were used to evaluate annual mortality between 1998 and 2018. Polynomial regressions were performed to assess annual changes in CHD-associated mortality rates by age groups.

**Results:**

During the 21-year study period, a total of 11,314 deaths were attributed to CHD with 50.9% of deaths in infants (age < 1 year) and 28.2% in neonates (age ≤ 28 days). The most frequent underlying CHDs associated with death were hypoplastic left heart syndrome (*n* = 1498, 13.2%), left ventricular outflow tract obstruction (*n* = 1009, 8.9%), atrial septal defects (*n* = 771, 6.8%), ventricular septal defects (*n* = 697, 6.2%), and tetralogy of Fallot (*n* = 673, 5.9%), and others (*n* = 6666, 58.9%). Among all patients, annual CHD-related mortality rates declined significantly between 1998 and 2010 (*p* < 0.0001), followed by a significant annual increase until 2018 (*p* < 0.0001). However, mortality rates in 2018 in all ages were significantly lower than in 1998.

**Conclusion:**

Mortality in CHD patients decreased significantly between 1998 and 2010, but a substantial number of deaths still occurred and even significantly increased in the last 3 years of the observation period particularly in neonates and infants. This renewed slight increase in mortality rate during the last years was influenced mainly by high-risk neonates and infants. Assessment of factors influencing the mortality rate trends in association with CHD in Germany is urgently needed. Obligatory nationwide registration of death cases in relation to surgical and catheter interventions in CHD patients is necessary to provide additional valuable data on the outcome of CHD.

**Graphical abstract:**

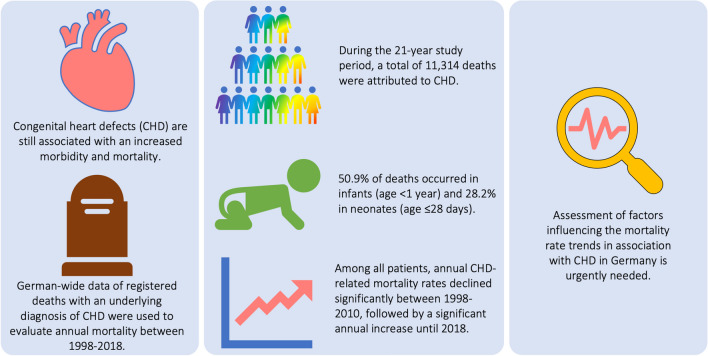

## Introduction

Congenital heart defects (CHD) are the most common congenital organ malformations in the newborn [1–4]. Management of complex rather than simple congenital heart and vascular malformation is still challenging and such malformations are still associated with an increased mortality and morbidity. Pre- and postnatal, as well as treatment-related risk factors, may contribute to the mortality of infants with CHD in early life. In complex CHDs with altered systemic and pulmonary perfusion, patent fetal connections like the patent ductus arteriosus and the foramen ovale provide a sufficient systemic and pulmonary perfusion resulting in a specious asymptomatic period immediately after birth [5–8]. Thus, prenatal and early postnatal diagnosis of severe cardiac and vascular malformations is mandatory to avoid altered systemic and pulmonary perfusion and thereby death in these neonates [6, 7, 9].

Depending on the severity of such cardiovascular defects, immediate postnatal surgery or catheter intervention may be required. Due to the immaturity of several physiological functions, which exhibit an adaptation and transition from fetal to neonatal life, surgical repair by using cardiopulmonary bypass and circulatory arrest in early life may represent an additional risk factor for myocardial failure, organ injury, and death [10, 11].

Representative data on nationwide mortality trends over time in different ages and types of CHDs in Germany during the last two decades are limited because of the lack of a mandatory registration by law of all CHD deaths in the established national research registry of CHD or in the quality assurance survey programs [12]. As part of the federal quality assurance for CHD, hospitals can report freely all born or treated cases with CHD. However, not all hospitals are members of this national registry and quality assurance survey and the registration is mainly voluntary. According to this voluntary approach, a nationwide complete registration and comprehensive coverage of all CHD cases treated, not treated, or died are not possible and may not be representative.

However, in Germany, every death case, independent of age, diagnosis, or location of death, must be officially documented with the underlying diagnosis of disease in and registered with the public health department by federal law. Related clinical and therapeutic circumstances are however not included in this obligatory death register. The data are controlled and completed by the health department of the different federal states before transferring the complete data sheet to the central federal statistic office. These data are compiled and stored by the Federal Statistical Office of Germany (DESTATIS, Wiesbaden, Germany) according to the International Statistical Classification of Diseases and Related Health Problems (ICD) and are freely accessible for research institutes for health and epidemiological research [13].

Data from other industrialized Western countries demonstrated a substantial decrease in mortality attributable to CHD [14]. According to the comprehensive registration and documentation of all deaths in Germany, it was possible to assess the number of all deaths attributed to CHDs in Germany over a period of 21 years. Thus, the aim of our study was to analyze the development of mortality rates resulting from CHD between 1998 and 2018 in Germany, and to compare our data to previously published reports.

## Methods

### Study design and population

In this study, we analyzed all deaths with an underlying ICD-10 diagnosis (2000–2018) and ICD-10 equivalent classification (1998–1999) of CHD in Germany. All relevant anonymized data were retrieved from the freely accessible database “The Information System of Federal Health Monitoring” [13]. The “Information System of Federal Health Monitoring” is a joint service by Robert Koch Institute (RKI, Berlin, Germany) and the Federal Statistical Office of Germany (DESTATIS, Wiesbaden, Germany). German-wide data with complete yearly information on deaths attributed to CHD throughout lifespan were included in the present analysis.

### Data collection

Annual data included in this analysis included the number of deaths with underlying congenital malformations of the heart and circulatory system (ICD-10: Q20–Q28), age at the time of death, and overall population in Germany.

### Outcome

Annual mortality rate per 1000 persons for a defined age group was calculated as the total number of deaths attributed to CHD divided by the total number of persons of the overall population in the particular age group in the respective year.

### Statistical analyses

Analysis was restricted to registered deaths attributed to a CHD diagnosis. We did not evaluate the underlying reasons of deaths, such as myocardial failure before/after surgical repair, because this data is not available in “Information System of Federal Health Monitoring” [13]. Moreover, stillbirths as well as miscarriages (https://www.bib.bund.de/DE/Fakten/Glossar/glossar.html?cms_lv2=1215792&cms_lv3=1215582, accessed 11/17/2022) because of severe CHD sickness were not documented and were not part of this analysis.

We performed polynomial regressions (with one, two, or three degrees) using annual mortality rate as outcome and year as the explanatory variable. The best model was chosen based on the lowest Akaike information criterion and the highest *R*^2^ value. Data were analyzed using R software, version 4.0.5. A *p*-value of < 0.05 was considered statistically significant.

## Results

**Fig. 1:**
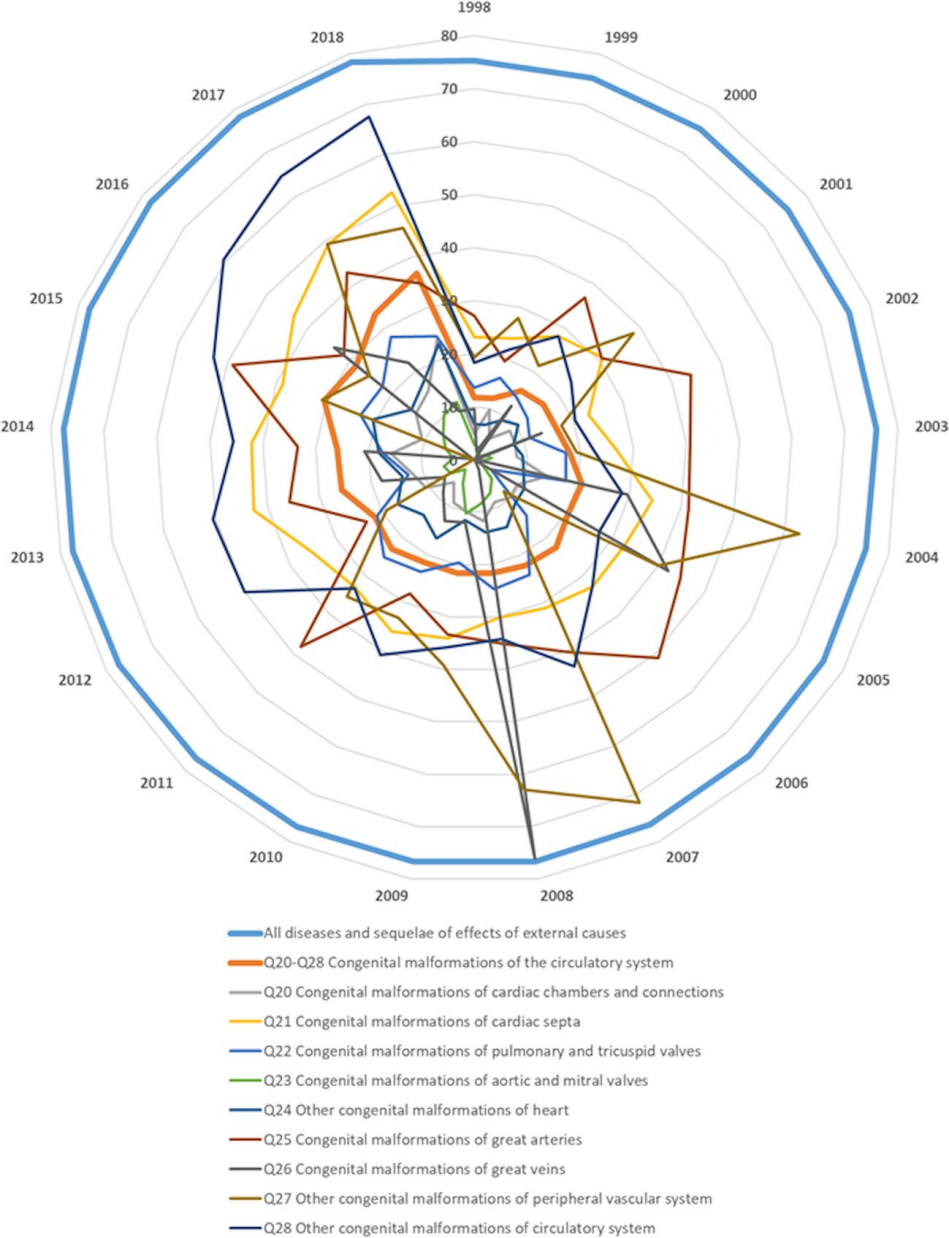
Mean age of the deceased in years of age from 1998 to 2018. Data are depicted for “all diseases and sequelae of effects of external cause” as well as for “congenital malformations of the circulatory system” (ICD-10 code: Q20–Q28)

During the 21-year period (1998–2018), a total of 11,314 deaths attributed to CHD occurred in Germany according to official death statistics. The age at the time of death ranged from the early neonatal period to more than 90 years of age; the mean age of the deceased is depicted in Fig. 1. Remarkably, over the studied period, 50.9% (*n* = 5756) of all death cases attributed to CHD were < 1 year of age, and 28.2% (*n* = 3187) of all death cases were neonates (≤ 28 days of age).

The most frequent underlying CHDs (Table 1) associated with death *in all age groups* (*n* = 11,314) were hypoplastic left heart syndrome (*n* = 1498, 13.2%), left ventricular outflow tract obstruction (*n* = 1009, 8.9%), atrial septal defects (*n* = 771, 6.8%), ventricular septal defect (*n* = 697, 6.2%)*,* tetralogy of Fallot (*n* = 673, 5.9%), and others (*n* = 6666, 58.9%).

**Table 1:** Prevalence of congenital heart defects (*CHD*) in newborns in Germany modified according to Lindinger et al. [3] in comparison to CHDs associated with mortality depicted as absolute and relative numbers

Cardiac malformation	Prevalence of CHDs in Germany (*n* = 7245)	CHDs associated with death *in all age groups* (*n* = 11,314)	CHDs associated with death < *1 year* of age (*n* = 5756)
VSD	3545 (48.9%)	697 (6.2%)	214 (1.9%)
ASD	1235 (17.0%)	771 (6.8%)	101 (0.9%)
PS/PA	508 (7.0%)	259 (2.3%)	145 (1.3%)
PDA	310 (4.3%)	239 (2.1%)	172 (1.5%)
CoA	264 (3.6%)	232 (2.1%)	97 (0.9%)
AVSD	200 (2.8%)	244 (2.2%)	142 (1.3%)
ToF	179 (2.5%)	673 (5.9%)	213 (1.9%)
AS	161 (2.2%)	1009 (8.9%)	260 (2.3%)
D-TGA	156 (2.5%)	374 (3.3%)	219 (1.9%)
HLHS	101 (1.4%)	1498 (13.2%)	1359 (12.0%)
DORV	76 (1.0%)	193 (1.7%)	120 (1.1%)
TAPVC	43 (0.6%)	82 (0.7%)	73 (0.6%)
TAC	33 (0.5%)	149 (1.3%)	107 (0.9%)
Ebstein anomaly	27 (0.4%)	243 (2.1%)	94 (0.8%)
PAPVC	26 (0.4%)	14 (0.1%)	6 (0.1%)
L-TGA	25 (0.3%)	37 (0.3%)	19 (0.2%)
IAA	22 (0.3%)	33 (0.3%)	28 (0.2%)
Others	232 (3.2%)	4567 (40.4%)	2387 (21.1%)

*AS* aortic stenosis, *ASD* atrial septal defect, *AVSD* atrioventricular septal defect, *CoA* coarctation of the aorta, *L-TGA* congenitally corrected transposition of the great arteries, *DORV* double outlet right ventricle, *HLHS* hypoplastic left heart syndrome, *IAA* interrupted aortic arch, *PAPVC* partial anomalous pulmonary venous connection, *PDA* patent ductus arteriosus, *PA* pulmonary atresia, *PS* pulmonary stenosis, *ToF* tetralogy of Fallot, *D-TGA* transposition of the great arteries, *TAPVC* total anomalous pulmonary venous connection, *TAC* truncus arteriosus communis, *VSD* ventricular septal defect

When considering only *deaths* < *1 year* of age (*n* = 5756), the most common underlying CHDs associated with death were hypoplastic left heart syndrome (*n* = 1359, 23.6%), left ventricular outflow tract obstruction (*n* = 260, 4.5%), transposition of the great arteries (*n* = 219, 3.8%), ventricular septal defect (*n* = 214, 3.7%), tetralogy of Fallot (*n* = 213, 3.7%), and others (*n* = 3491, 60.6%).

**Fig. 2:**
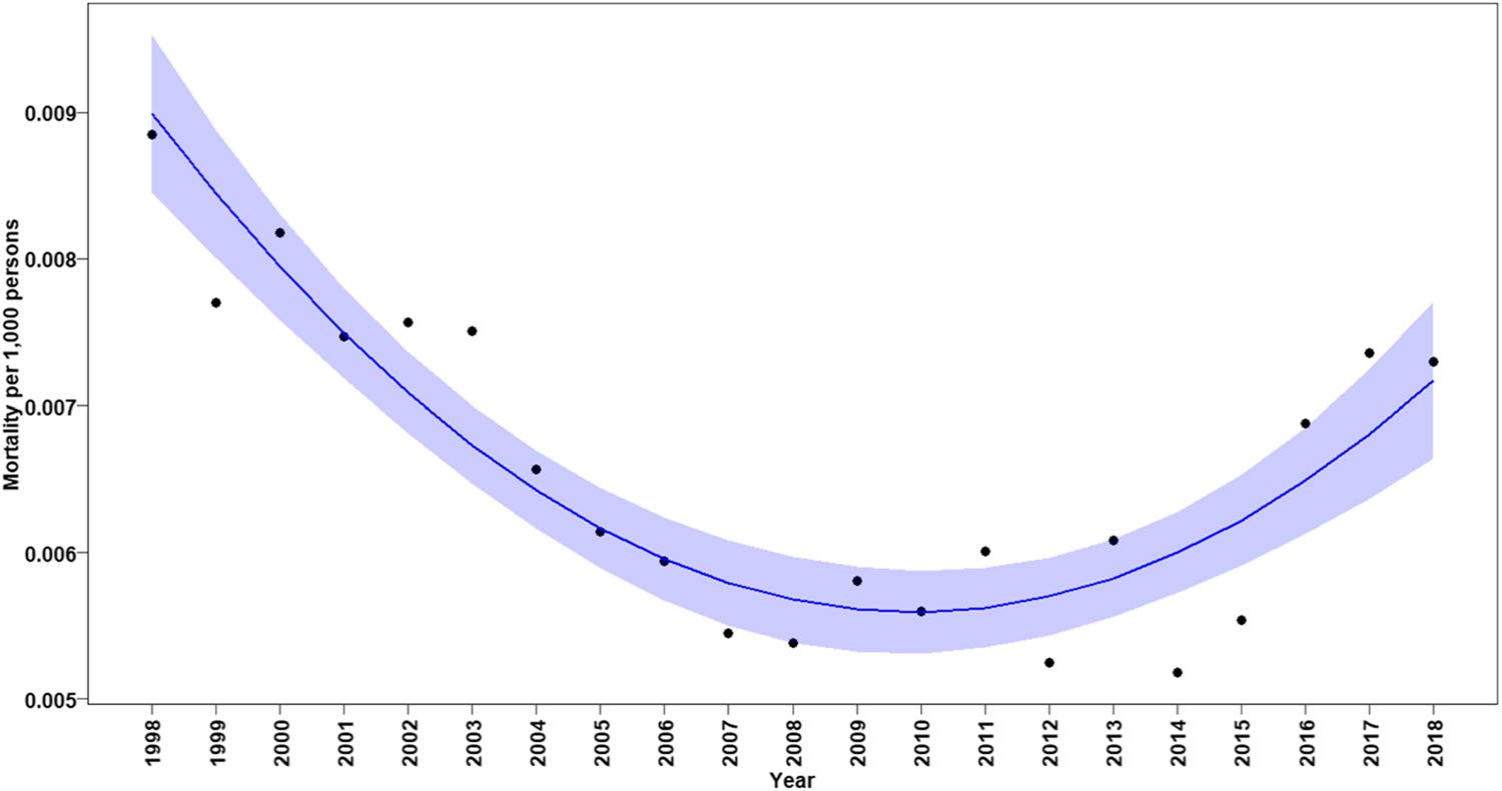
Annual mortality rates associated with any CHD in all ages. Black dots show observed mortality rate per year. Blue line shows the predicted mortality rate curve along with its 95% confidence interval based on a quadratic polynomial regression

**Fig. 3:**
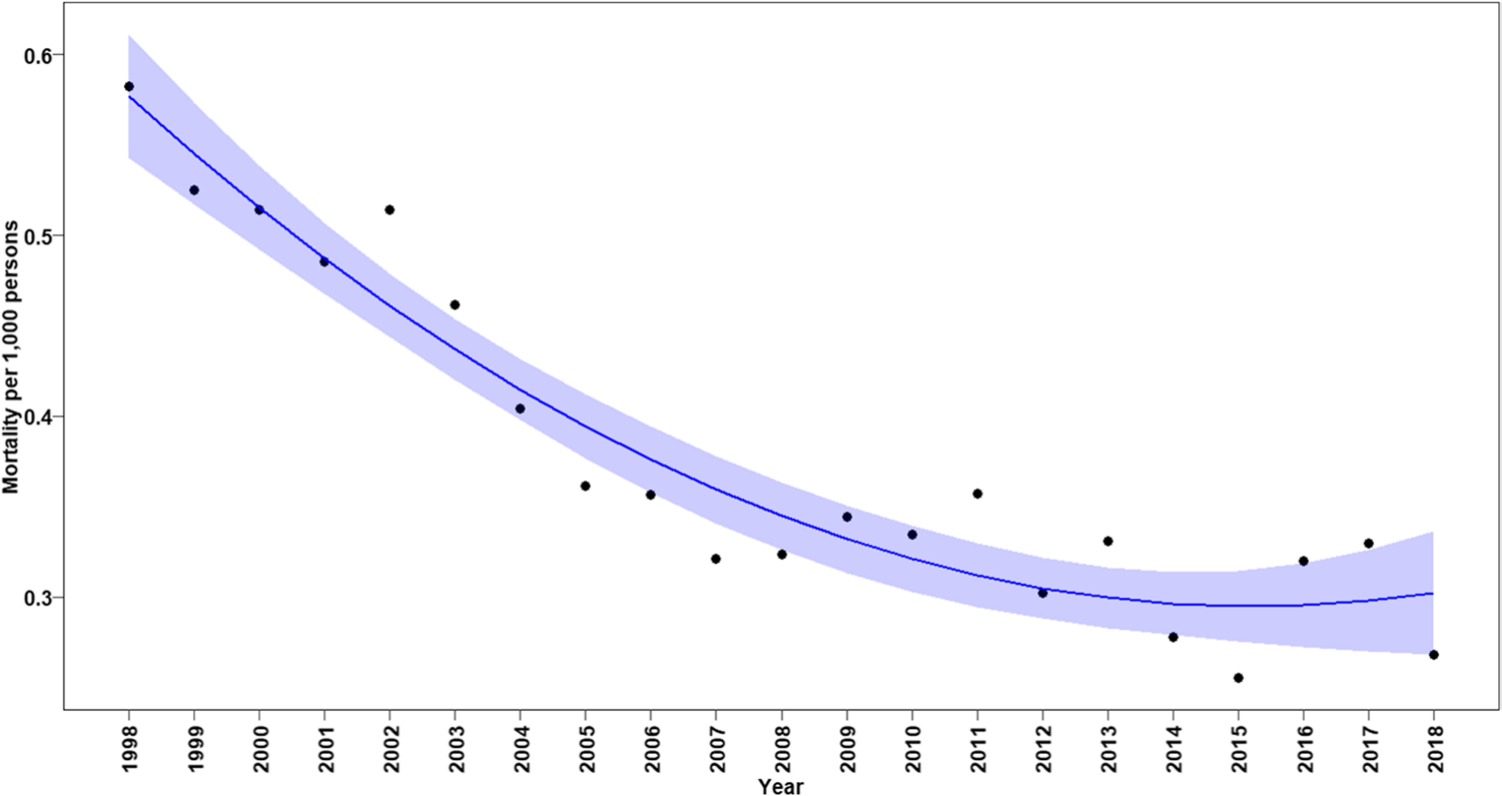
Annual mortality rate associated with any CHD in infants (age < 1 year). Black dots show observed mortality rate per year. Blue line shows the predicted mortality rate curve along with its 95% confidence interval from a quadratic polynomial regression

Among all patients, the annual CHD-related mortality rates declined significantly between 1998 and 2010 (annual change in mortality rate: –0.00057, *p* < 0.0001, Fig. 2). However, from 2010 until 2018, a renewed significant gradual increase in mortality rates in all age groups was noted (annual change in mortality rate: 0.00002, *p* < 0.0001, Fig. 2). When considering CHD-related mortality rates according to age in early infancy, we found a continuous significant reduction in mortality rates in all neonates and infants under the age of 1 year until 2015. However, also in this age group, the trend of decreased mortality was sustained and a renewed increase in mortality rate was found (annual change in mortality rate between 1998 and 2015: –0.033, *p* < 0.0001 and annual increased change in mortality rate between 2015 and 2018: 0.00095, *p* = 0.00013, Fig. 3).

**Fig. 4:**
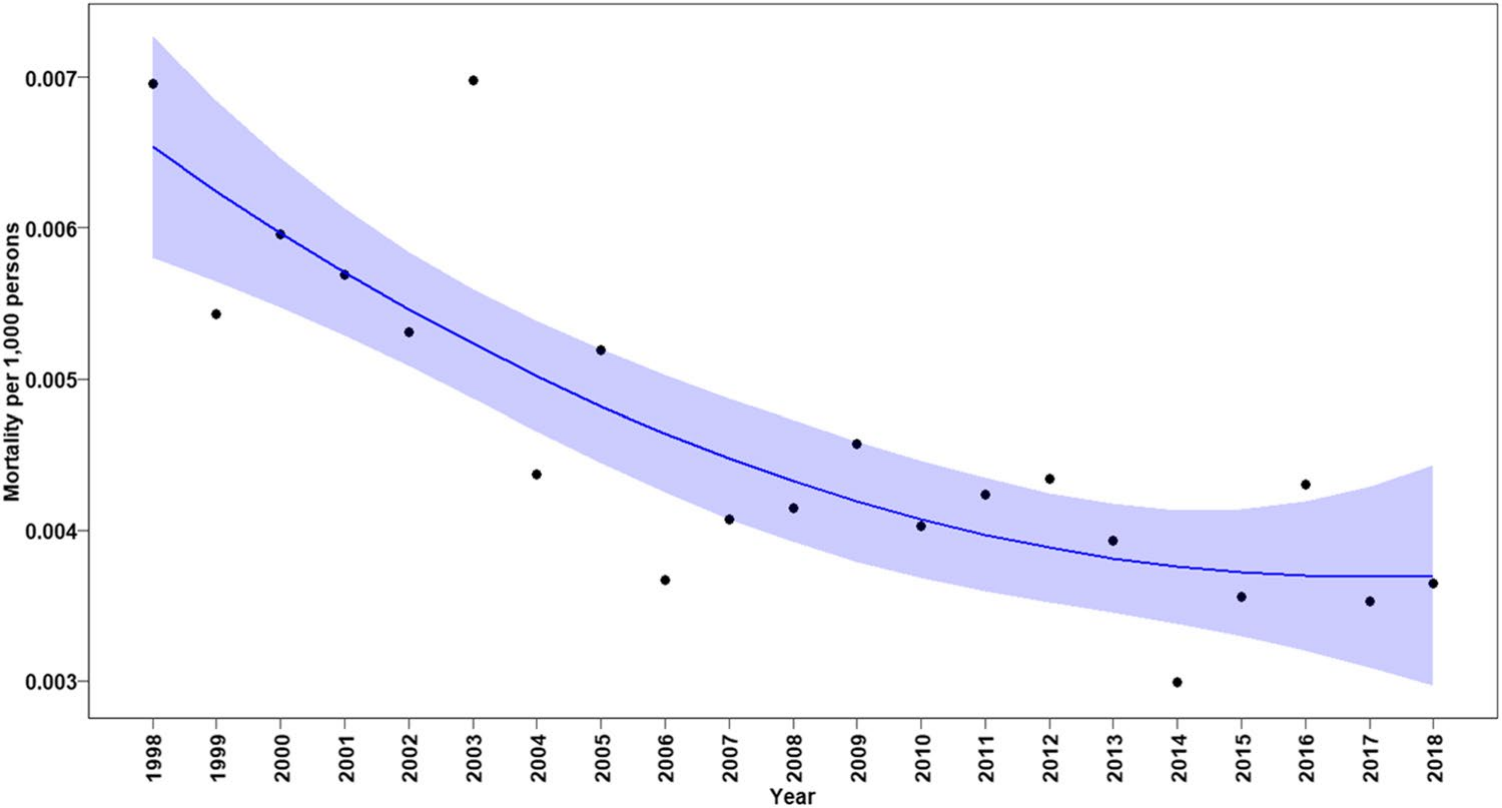
Annual mortality rate associated with any CHD in persons aged 1 to 20 years. Black dots show observed mortality rate per year. Blue line shows the predicted mortality rate curve along with its 95% confidence interval from a quadratic polynomial regression

Similarly, in the patients beyond the age of 1 year until the age of 20 years, there was a significant decline in the rate of mortality from 1998 to 2014 (annual change in mortality rate between 1998 and 2014: –0.00030, *p* = 0.00272, Fig. 4). After 2014, tendential but not significant changes in the annual mortality rates were observed (*p* = 0.07370).

**Fig. 5:**
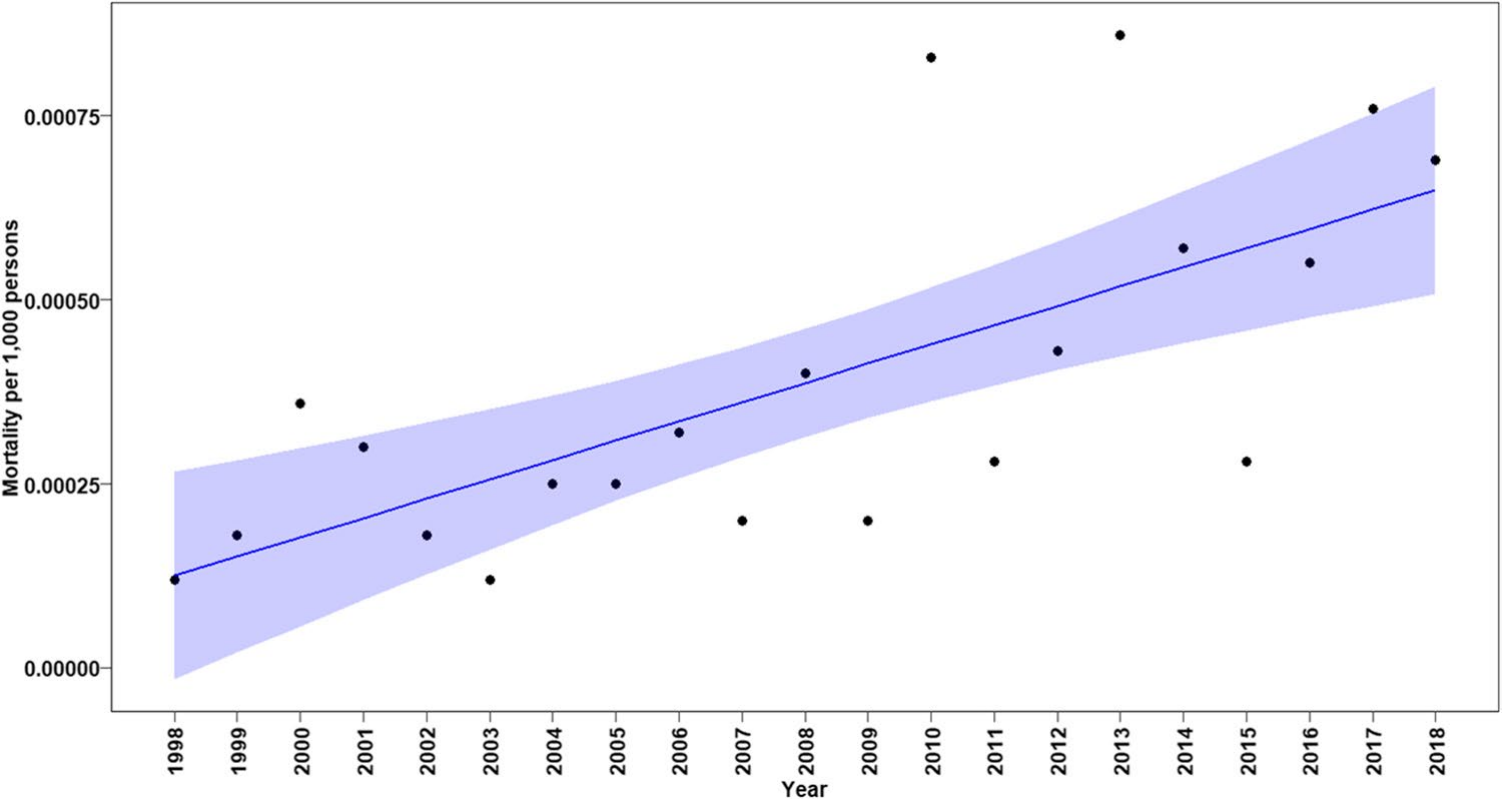
Annual mortality rate from hypoplastic left heart syndrome in persons aged 1 to 20 years. Black dots show observed mortality rate per year. Blue line shows the predicted mortality rate curve along with its 95% confidence interval from a linear regression

However, despite being the second most frequent underlying CHD associated with death in all patients, mortality rates attributed to left ventricular outflow tract obstruction did not change over time (data not depicted). In contrast, in patients aged 1 to 20 years, the hypoplastic left heart syndrome-related mortality rates increased annually (annual change in mortality rate: 0.00003, *p* < 0.0001, Fig. 5) throughout the observation period. This observed increase contributes substantially to the increase of mortality rates between 2010 and 2018 in all ages (Fig. 2).

**Fig. 6:**
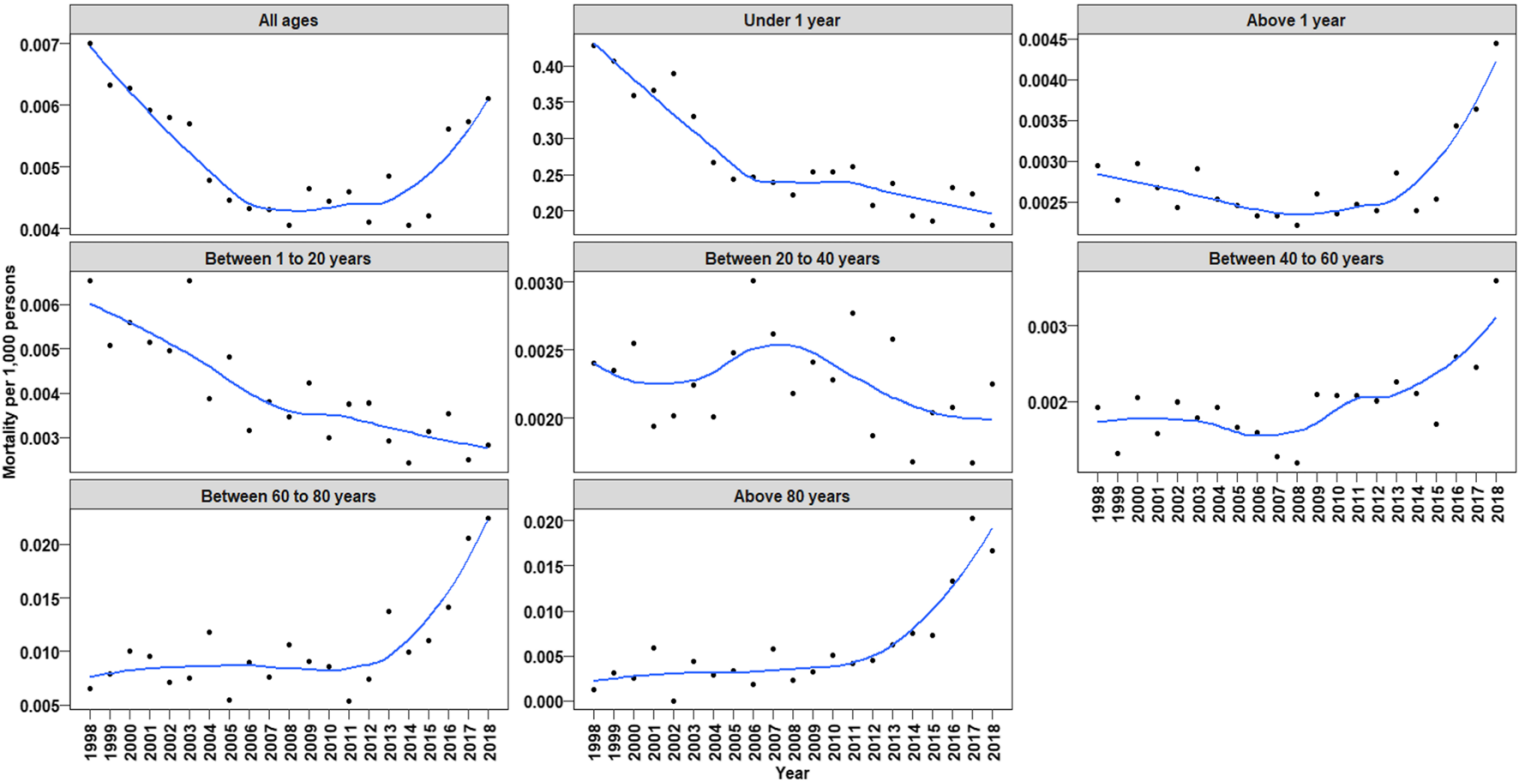
Annual mortality rates associated with any CHD for different age groups. Plots show real data fitted with smoothing method LOESS (locally estimated scatterplot smoothing)

For comparison of CHD-associated mortality rates, Table 2 provides an overview of the overall mortality rates in Germany between 1998 and 2018. Moreover, in Fig. 6, trends in mortality rates in different age groups for patients with any CHD are depicted.

**Table 2:** Mortality rates in Germany: Overall mortality rates of the general population and overall mortality rates in patients with congenital heart defect (CHD) are depicted per 1000 residents in Germany. Overall mortality rates in infants < 1 year of age and overall CHD mortality in infants < 1 year of age are depicted per 1000 residents < 1 year of age in Germany

Year	Overall mortality	CHD mortality	Overall mortality in infants < 1 year of age	CHD mortality in infants < 1 year of age
1998	10.4	0.009	4.7	0.582
1999	10.3	0.008	4.5	0.525
2000	10.2	0.008	4.4	0.514
2001	10.1	0.007	4.3	0.485
2002	10.2	0.008	4.2	0.514
2003	10.3	0.008	4.2	0.461
2004	9.9	0.007	4.1	0.404
2005	10.1	0.006	3.9	0.362
2006	10.0	0.006	3.8	0.357
2007	10.1	0.005	3.9	0.321
2008	10.3	0.005	3.5	0.323
2009	10.4	0.006	3.5	0.344
2010	10.5	0.006	3.4	0.335
2011	10.6	0.006	3.7	0.357
2012	10.8	0.005	3.3	0.302
2013	11.1	0.006	3.3	0.331
2014	10.7	0.005	3.2	0.278
2015	11.3	0.006	3.2	0.255
2016	11.1	0.007	3.4	0.320
2017	11.3	0.007	3.3	0.330
2018	11.5	0.007	3.2	0.268

## Discussion

Since 1998, all death cases are obligatorily documented by federal law from all federal states and are available for different nationwide epidemiological and health studies. Our epidemiological study revealed important valuable nationwide trends in mortality rate associated with CHD in all age groups over a 21-year period in Germany. We found that CHD mortality in infants is still the highest in comparison to all other age groups and contributed substantially to the overall mortality associated with CHD in Germany.

While CHD-associated mortality in Germany decreased significantly during the first decade of the studied period, particularly in infants under the age of 1 year, a slight but significant renewed increase in mortality was found in the last 8 years (2010–2018). The predominantly high-risk group for death in both periods remained neonates and infants under the age of 1, particularly those with hypoplastic left heart syndrome, one of the more complex and severe CHDs, which contributes significantly to the overall mortality in this cohort. Thus, the survival of neonates and infants < 1 year of age born with CHD in the last two decades strongly influenced the overall mortality trends in patients with CHD. The majority of infants requiring surgical repair in early life are those with severe CHDs, like hypoplastic left heart syndrome and transposition of great arteries. The data tentatively implicate that the medical management and care before and after cardiac surgery of such high-risk neonates has been improved significantly in this period.

The documented unexpected change in mortality trends between 2010 and 2018, however, is surprising and needs further nationwide evaluation of the quality insurance of all pediatric cardiac centers in Germany. Mortality rates in patients with hypoplastic left heart syndrome seem to contribute to the renewed increase in mortality rates. We speculate that neonates and infants with hypoplastic left heart syndrome, who previously had palliative surgery, now result in increasing numbers of adults with HLHS and increased mortality in adults with HLHS.

Especially, since 2014 in infants under the age of 1 year, a slight but significant increase in their mortality rate can be observed. One factor seems to be a renewed increase in the mortality rate in the high-risk group of children with hypoplastic left heart syndrome. To explain such phenomena, however, more reliable data from different centers, including statistical assessments of surgical and interventional catheter procedures rather than the diagnosis alone, is necessary. Unfortunately, the surgical and interventional procedure codes were not available from the database “The Information System of Federal Health Monitoring” [13] and were therefore not included in this analysis. Interestingly, mainly in adults, mild CHDs such as atrial septal defect and ventricular septal defect seemed to contribute to mortality in this cohort (Fig. 1). Several studies have documented increased trends in mortality in adult age even in patients with mild CHD [15, 16].

Similar to other high-income countries, gradual improvements in early diagnostic, surgical as well as interventional management on the field of neonatal cardiology and surgery contributed to the significant decrease in mortality rate associated with CHD in the documented time intervals. Improvements in the overall survival of infants and children were noticed at national and international levels during the last decades [17, 18]. And in the cohort of CHD patients, improvements of survival have also been reported [14, 19–23]. Gilboa et al. [19] found a reduction of mortality in patients with CHD in the USA. The reduction of mortality ranged from 17.3% to 36.1% between 1999 and 2006 depending on the observed age class what is somehow comparable with our results, and Lopez et al. [14] even reported a decrease in CHD mortality of 39.4% (1999–2017). However, in our cohort including nationwide documented cohorts, the improvement of the overall CHD-mortality (1998 versus 2018: 16.5%) and the mortality in infants with CHD under 1 year of age (1998 versus 2018: 54.6%), were higher than the reported data by Gilboa and colleagues [19]. The percentage of newborns within the group of infants dying with underlying CHD was not evaluated in this study. Though Lopez et al. [14] found infant mortality to cause 47.7% of all deaths attributable to CHD, Khoshnood et al. [20] found neonatal CHD mortality to contribute with 60% to overall infant deaths and Pérez-Lescure Picarzo et al. [21] found an even higher mortality of 79.2% resulting from congenital heart and vessels defects in neonates. The Congenital Heart Disease Collaborators [22] estimated that approximately 69% of congenital heart defect deaths in 2017 occurred < 1 year of age.

Differences in the observed regions and time intervals might in part explain the different results concerning the improvements of mortality in infants with CHD between our data and other studies, but a recent study from the USA demonstrated a similar pattern of mortality rates (1999–2017) [14, 22]. Moreover, by establishing regional pediatric cardiac centers during the observed period, it is highly likely that improvements in diagnostics and therapy of CHD in Germany occurred.

However, it has to be kept in mind that in some regions/countries CHD mortality is still increasing [22, 24]. Cabral et al. [24] describe in their publication a linear growth of mortality in infants by approximately 0.5/year from 1996 to 2016.

Although a significant improvement of the overall survival in patients with CHD has been achieved in the last decades (Fig. 1), the management of severe CHD in early life remains challenging. Neonates and infants with severe CHD such as hypoplastic left heart syndrome, transposition of the great arteries, or tetralogy of Fallot still need highly specialized intensive care units before, during, and after surgery. Surgical repair and palliative surgical therapy require the use of cardiopulmonary bypass and deep hypothermic circulatory arrest, which put these immature vulnerable neonates and infants on high risk for myocardial failure as well as injury of other organs. Certainly, contributing factors for the increase of survival in this vulnerable cohort are improvements in the field of pediatric cardiac surgery, catheter interventions, and pediatric cardiac intensive care [25]. Moreover, improved early prenatal and postnatal detection of CHD, resulting in planning the birth in perinatal centers with immediate care by pediatric cardiac centers, may have also contributed to the reduction of death in these patients [26–31].

The significant gradual renewed increase in mortality rates, particularly in neonates and infants during the first year of life, who require immediate therapeutic interventions, is unexpected and needs further assessment and evaluation. The latest data until 2021 demonstrate a sustained increased mortality rate in this cohort (data not depicted) which implies that this is no transient phenomenon. We speculate that several medical, regulative, and socioeconomic factors, which occurred in Germany during the time intervals, may have contributed to the change in the mortality trends during the assessed time periods. Since the mid of the last decade, a reduction in pediatric intensive care capacity occurred, resulting in a reduction of intensive care beds and delaying of important surgical procedures. Unfortunately, this situation in nearly all pediatric intensive care units persists until today which dramatically influences the capacity of pediatric cardiac surgery [32]. The main reason for this reduction of intensive care capacities is mainly a lack of number of intensive nurses.

In 2006 and 2017, “The Federal Joint Committee” has released guidelines concerning nursing of premature and newborns at the ICU, which has led and still leads to a shift of nursing staff into neonatal intensive care units. This, in turn, results in a lack of nursing staff in highly specialized pediatric cardiac intensive care units (https://www.dhzb.de/presse/news/detailansicht-meldungen/ansicht/pressedetail/pflegenotstand-in-der-kinderherzmedizin, accessed 11/17/2022). Moreover, in 2010, “The Federal Joint Committee” also published guidelines regarding the quality assurance of cardiac surgery care in children (https://www.g-ba.de/downloads/39-261-1102/2010-02-18-Kinderherzchirurgie-RL-Erstfassung_BAnz.pdf, accessed 11/17/2022). These guidelines specify in detail requirements regarding staffing, infrastructure, and organization of pediatric cardiac centers. On the one hand, the aforementioned guidelines ensure a highly specialized care of these patients but on the other hand, it has resulted in a reduction of pediatric cardiac centers dealing with patients with CHDs. Although, these guidelines became operative in 2010, it has taken some time until they were fully implemented, which might explain why the renewed increase in mortality rates started in 2010 and increased until 2018, when these guidelines were fully operative [33].

In line with the paradoxical increased mortality rates in CHD patients from 2010 to 2018, we also observed an increase of live-born children during the same period in Germany. Improvements in neonatal and perinatal medicine have led to improved survival of preterm born babies [34]. The risk of a preterm delivery is two- to threefold higher in neonates with CHD and the other way round the risk of having a CHD is higher in premature infants than in their term counterparts [34, 35]. Although mortality in preterm neonates with CHD decreased over the last decades among others due to improvements in neonatal cardiac surgery, they have the highest mortality compared to their counterparts without CHD [36, 37]. In the USA, CHD and prematurity are the main reasons for infant mortality [38]. Altogether, improvements in perinatal care might cause increased survival of fetuses but might also postpone death into the neonatal period especially in children with critical CHDs.

Furthermore, during the time period of the observed results, migration, particularly from crisis regions, was increased. Inequalities in utilization of medical care by immigrants have been documented, which was limited by socioeconomic factors and other reasons such as lack of information, as well as language barriers [39, 40].

### Limitations

The aim of this study was to evaluate nationwide age-related mortality rates in association with CHD, rather than the analysis of mortality rates in relation to surgical or interventional procedures in different regions in Germany. Collecting robust and reliable nationwide complete datasets concerning the pre- and postoperative management, as well as all surgical and interventional procedures, was not feasible. Thus, statistical analysis in this regard was deliberately waived. However, procedure-related and regional analysis of mortality rates should be ideally prospectively assessed in future studies.

Moreover, in the database, “The Information System of Federal Health Monitoring”[13] the underlying CHD is listed as cause of death. However, misreporting of underlying mild CHDs as the leading cause of death might have unduly inflated the mortality rates attributed to mild CHDs. In addition, the quality of death certificates may vary substantially depending on the training and experience of the physician and lack of time to properly fill in these documents [41]. It is also important to note that the ICD has rules for determining a single underlying cause of death in each case but does not take into account the possibility of multiple contributing, intermediate, or immediate causes of death [42]. Furthermore, it cannot be excluded that errors occurred during the ICD encoding process. The ICD codes do not differentiate simple and complex forms of CHDs with multiple cardiac malformations. As a consequence, it is possible that a mild co-pathology but not the leading cardiac pathology was encoded, which would also unduly inflate the mortality rates attributed to mild CHDs.

Moreover, we did not assess important covariates in our cohort, e.g., prematurity and underlying genetic syndromes. Of note, the main aim of our study was to assess changes in mortality rates over time. The relatively higher mortality rates seen in association with mild CHD in all patients occur rather in the adult age. Several reports have also demonstrated higher morbidity and mortality in these patients particularly in those without therapy at younger ages. This might explain why atrial septal defects and ventricular septal defects, the most common CHDs, are the 3rd and 4th most frequent defects associated with mortality in all age groups in this study.

Obligatory rather than optional registration of relevant medical data, including procedure-related therapeutic intervention/surgery, pseudonymous personal data, and regional centers, is necessary to evaluate results and quality assurance measures in patients undergoing complex cardiac medical care in an even more comprehensive way.

Although quite unlikely, there is a possibility of changes in coding of CHDs over time secondary to changes in reimbursement that may have to some degree influenced our data.

### Conclusion

We conclude that the mortality in CHD patients substantially decreased over the last 21 years, but a substantial number of deaths still occurs particularly in infants < 1 year of age as well as neonates. The renewed slight increase in mortality rate throughout the lifespan during the last years is influenced mainly by high-risk infants with hypoplastic left heart syndrome. Mild common CHDs may also contribute to mortality rates in adult age. A nationwide obligatory registry recording all death cases primarily attributable to the underlying CHD as well as in relation to surgical and catheter therapeutic interventions in CHD patients would be helpful in providing valuable and comprehensive data on the outcome of CHD throughout lifespan.

## Data Availability

All relevant anonymized data used in this study were retrieved from the freely accessible database “The Information System of Federal Health Monitoring”. The “Information System of Federal Health Monitoring” is a joint service by Robert Koch Institute (RKI, Berlin, Germany) and the Federal Statistical Office of Germany (DESTATIS, Wiesbaden, Germany).
